# A GCDGC-specific DNA (cytosine-5) methyltransferase that methylates the GCWGC sequence on both strands and the GCSGC sequence on one strand

**DOI:** 10.1371/journal.pone.0265225

**Published:** 2022-03-21

**Authors:** Yoshikazu Furuta, Fumihito Miura, Takahiro Ichise, Shouta M. M. Nakayama, Yoshinori Ikenaka, Tuvshinzaya Zorigt, Mai Tsujinouchi, Mayumi Ishizuka, Takashi Ito, Hideaki Higashi

**Affiliations:** 1 Division of Infection and Immunity, International Institute for Zoonosis Control, Hokkaido University, Sapporo, Japan; 2 Department of Biochemistry, Kyushu University Graduate School of Medical Sciences, Fukuoka, Japan; 3 Laboratory of Toxicology, Department of Environmental Veterinary Sciences, School of Veterinary Medicine, Hokkaido University, Sapporo, Japan; 4 Water Research Group, Unit for Environmental Sciences and Management, North-West University, Potchefstroom, South Africa; Universität Stuttgart, GERMANY

## Abstract

5-Methylcytosine is one of the major epigenetic marks of DNA in living organisms. Some bacterial species possess DNA methyltransferases that modify cytosines on both strands to produce fully-methylated sites or on either strand to produce hemi-methylated sites. In this study, we characterized a DNA methyltransferase that produces two sequences with different methylation patterns: one methylated on both strands and another on one strand. M.BatI is the orphan DNA methyltransferase of *Bacillus anthracis* coded in one of the prophages on the chromosome. Analysis of M.BatI modified DNA by bisulfite sequencing revealed that the enzyme methylates the first cytosine in sequences of 5ʹ-GCAGC-3ʹ, 5ʹ-GCTGC-3ʹ, and 5ʹ-GCGGC-3ʹ, but not of 5ʹ-GCCGC-3ʹ. This resulted in the production of fully-methylated 5ʹ-GCWGC-3ʹ and hemi-methylated 5ʹ-GCSGC-3ʹ. M.BatI also showed toxicity when expressed in *E*. *coli*, which was caused by a mechanism other than DNA modification activity. Homologs of M.BatI were found in other *Bacillus* species on different prophage like regions, suggesting the spread of the gene by several different phages. The discovery of the DNA methyltransferase with unique modification target specificity suggested unrevealed diversity of target sequences of bacterial cytosine DNA methyltransferase.

## Introduction

The role of DNA modification in bacteria was long believed to be a part of the cell defense mechanism by restriction-modification systems, but it is now known to have other important roles in regulation of gene expression and phenotype of cells [1–3], which include regulation of DNA mismatch repair [4,5], DNA replication [6,7], and virulence [8–10]. The genome-wide pattern of methylation, or methylome, is known to be regulated by various mechanisms such as horizontal transfer of DNA methyltransferase (MTase) with mobile genetic elements [11], exchange of target recognition domain of MTase by genome rearrangement [12,13], and switching of MTase activity by phase variation [14]. A gene of bacterial MTase usually exists together with a paired gene coding restriction enzyme but is sometimes present in the genome without such a paired gene. Such an MTase gene is called an orphan MTase [15–17]. MTase cannot be an active part of cell defense without a paired restriction enzyme; thus, orphan MTases are expected to have other functions which is important enough to be maintained in a genome.

MTases were also found to be possessed by mobile genetic elements such as bacteriophages, plasmids, and transposons [11]. Many bacterial species possess restriction-modification systems using restriction enzymes to digest intruding DNA such as phages and plasmids, while the self-genomic DNA is protected with methylation by MTase. Mobile genetic elements that obtain MTase genes can protect their DNA and avoid digestion by restriction enzymes when they invade host cells. These MTases on mobile genetic elements are frequently found as orphan MTases, probably because of little or no advantage of restriction enzymes for mobile genetic elements.

Bacterial DNA methyltransferases are known to produce one of three kinds of DNA modification: N^6^-methyladenine (6mA), N^4^-methylcytosine (4mC), and 5-methylcytosine (5mC) [18]. 5mC is the most prevalent in the methylome of eukaryotic cells, while it is relatively less prevalent in bacteria compared to 6mA. For genome-wide analysis of DNA modification patterns, bisulfite sequencing is used for detection of 5mC [19,20] while SMRT sequencing technology by a PacBio sequencer is used for detection of 6mA and 4mC [21]. The latter was mainly used for bacterial methylome analysis partly because of the higher prevalence of MTases producing 6mA or 4mC than those producing 5mC [22] and also partly because the long reads produced by PacBio sequencers can be also used for the construction of a complete genome sequence in addition to methylome analysis. Due to the lower sensitivity of PacBio sequencers for 5mC, bacterial MTases that produce 5mC were less characterized compared to those produce 6mA and 4mC.

*Bacillus anthracis* is a gram-positive bacterial species that causes anthrax in humans and other animals [23]. In this study, we characterized the MTase possessed by *B*. *anthracis* which is an orphan MTase found on a prophage region. Its recognition sequence was previously speculated in several studies of its sequence [24–26], but has not yet been confirmed by experiments. Our findings revealed that the MTase, referred to as M.BatI hereafter, has unique activity of producing two sequences with different methylation patterns: one fully-methylated and another hemi-methylated.

## Materials and methods

### Strains and plasmids

Bacterial strains used in this study (listed in **Table 1**) were cultured in lysogeny broth (LB) at 37°C under aerobic conditions with appropriate antibiotics when required. A map of the chromosome was plotted using Circos [27].

**Table 1:** Strains.

Strains	Feature	Reference
*B*. *anthracis*		
34F2	*Bacillus anthracis* vaccine strain	[28]
BYF10027	34F2 *ΔbatIM*	This study
*E*. *coli*		
SM10	*thi thr leu tonA lacY supE recA*::RP4-2-Tc::Mu Km	[30]
S17-1	F- *thi pro hsdR hsdM recA* [RP4-2 Tc::Mu Km::Tn7 (Tp Sm)]	[30]
NEB 10-beta	Δ(*ara*-*leu*) *7697 araD139 fhuA* Δ*lacX74 galK16 galE15 e14*- ϕ*80*d*lacZ*Δ*M15 recA1 relA1 endA1 nupG rpsL* (StrR) *rph spoT1* Δ(*mrr*-*hsdRMS*-*mcrBC*)	New England Biolabs
ER2796	λ- *fhuA2 Δ(lacZ)r1 glnV44 mcr-62 trp-31 dcm-6 zed-501*::*Tn10 hisG1 argG6 rpsL104 dam-16*::*Kan xyl-7 mtlA2 metB1 (mcrB-hsd-mrr)114*::*IS10*	[32]
BYF823	ER2796 pZE31-*tetR*	This study
BYF822	ER2796 pZE31-*tetR*-*batIM*(WT)	This study
BYF826	ER2796 pZE31-*tetR*-*batIM*(A98V)	This study
BYF827	ER2796 pZE31-*tetR*-*batIM*(D75N)	This study
BYF867	ER2796 pZE31-*tetR*-*batIM*(C86G)	This study

*B*. *anthracis* 34F2 [28] with *batIM* gene deletion was constructed using the markerless allelic exchange strategy [29]. Flanking sequences of the *batIM* gene on the chromosome were amplified for 500 bp each by PCR from genomic DNA of *B*. *anthracis* 34F2 and inserted into pRP1028 [29] using Gibson assembly master mix (New England Biolabs, Ipswich, MA, USA). The constructed plasmid was transformed into *E*. *coli* SM10 [30] and introduced to *B*. *anthracis* 34F2 by conjugation. The strain with the plasmid integrated into the target site was selected and conjugated with pRP1099 from *E*. *coli* S17-1 [30] to induce a double strand break on the target site within the integrated region by I-SceI. A strain with *batIM* gene deletion was selected, resulting in strain BYF10027.

Plasmids for M.BatI induction in *E*. *coli* was constructed (**S1 Fig** and **S1 File**). First, the *tetR* gene was inserted into pZE31 [31] for tight regulation when uninduced, resulting in the construction of pZE31-*tetR*. Next, a wildtype *batIM* gene was amplified from genomic DNA of *B*. *anthracis* 34F2 and inserted into pZE31-*tetR* using Gibson assembly master mix (New England Biolabs, Ipswich, MA, USA), resulting in pZE31-*tetR*-*batIM* (WT). A C86G mutation was introduced into the pZE31-*tetR*-*batIM* (WT) by site-directed mutagenesis. *E*. *coli* NEB 10-beta (New England Biolabs, Ipswich, MA, USA) was used for all plasmid construction procedures before transforming constructed plasmids into *E*. *coli* ER2796 [32] for use in further experiments.

For isolation of *batIM* variants with methylation activity but lower toxicity, wildtype *batIM* was inserted in pZE31-*tetR* as above but selected on the LB agar plate with anhydrotetracycline (aTc) 100 ng/ml to induce M.BatI. Survived colonies were collected and plasmids were extracted using the QIAprep Spin Miniprep kit (QIAGEN, Hilden, Germany). To remove the plasmids with *batIM* variants that lost methylation activity, the plasmids were first digested with Fnu4HI (New England Biolabs, Ipswich, MA, USA) at 37°C for 1 h, then purified using the MinElute PCR Purification kit (QIAGEN, Hilden, Germany), and reacted with ExoV (RecBCD) (New England Biolabs, Ipswich, MA, USA) at 37°C for 30 min to remove linearized plasmids. Note that the plasmid possesses 17 Fnu4HI sites, thus methylation activity high enough to methylate all the sites are required to avoid cleavage at unprotected sites and subsequent digestion by ExoV (RecBCD). EDTA was added to 11 mM EDTA (pH 8.0) and incubated at 70°C for 30 min to inactivate enzymes, then purified using MinElute PCR Purification kit (QIAGEN, Hilden, Germany). The plasmids were then transformed into an *E*. *coli* NEB 10-beta (New England Biolabs, Ipswich, MA, USA). Plasmids were extracted from the transformants and subjected to Sanger sequencing to confirm their mutations. Single mutations of A98V and D75N were detected independently. The genes of *batIM* mutants were amplified by PCR from isolated plasmids and inserted in pZE31-*tetR*, transformed into NEB 10-beta (New England Biolabs, Ipswich, MA, USA), and used in further experiments.

### Isolation and digestion of genomic DNA

For *B*. *anthracis* strains, overnight cultures were diluted 100-fold into 25 ml LB and cultured for 8 h. Genomic DNA was extracted using QIAamp PowerFecal DNA Kit (QIAGEN, Hilden, Germany). For *E*. *coli* strains, overnight cultures were diluted 100-fold into 20 ml LB and cultured for 3 h. Induction was done by addition of aTc to 100 ng/ml for an hour before genomic DNA was extracted with QIAamp PowerFecal DNA Kit (QIAGEN, Hilden, Germany).

For DNA digestion experiments, 200 ng of genomic DNA was challenged by Fnu4HI, HaeIII, or MspI (New England Biolabs, Ipswich, MA, USA) at 37°C for an hour, followed by visualization using electrophoresis. All extractions and digestions were conducted in biological triplicate.

### Analysis of 5mC and C by UPLC-MS/MS system

To digest genomic DNA into nucleotide monomers, 200 ng of genomic DNA was treated with DNA Degradase Plus (Zymo Research, Irvine, CA, USA) at 37°C for 2 h, followed by filtering using 0.22 μm Millex Syringe Filters (Merck Millipore, Burlington, MA, USA) [33]. The separation of 5-methyl-2ʹ-deoxycytidine (5mdC) and 2ʹ-deoxycytidine (dC) was performed on a UHPLC PEEK column Inert Sustain Amide (2.1 mm × 50 mm, Φ1.9um; GL Science, Tokyo, Japan) at a flow rate of 1.0 ml/min and a temperature of 60°C. Mobile phase A was H_2_O containing 0.1% formic acid and mobile phase B was acetonitrile containing 0.1% formic acid. The following gradient program was applied: t = 0–1 min: 95% B; t = 7 min: 5% B; and t = 8 min: 5% B. The injection volume was 1 μl. Detection was performed using positive electrospray ionization. The triple quadrupole was operated in multiple reaction monitoring mode by monitoring of a quantifier and qualifier summarized in **Table 2**. Quantification of 5mdC and dC was achieved by external calibration using a standard curve built from calibration points at 10, 30, 50, 100, 200, and 300 nM. Molecular standards of 5mdC and dC were purchased from FUJIFILM Wako Pure Chemical Corporation (Osaka, Japan) and Tokyo Chemical Industry (Tokyo, Japan), respectively.

**Table 2:** Conditions of UPLC-MS/MS.

Compound Name	Retention time (min)	Precursor Ion (m/z)	Product Ion (m/z)	Collision Energy (V)	
5-Methyl-2ʹ-deoxycytidine	2.47	242.3	126.1	8	Quantifier
5-Methyl-2ʹ-deoxycytidine	2.47	242.3	109.1	44	Qualifier
2ʹ-Deoxycytidine	2.57	228.2	112.1	12	Quantifier
2ʹ-Deoxycytidine	2.57	228.2	95.1	44	Qualifier

### Whole-genome bisulfite sequencing

The preparation of libraries for shotgun bisulfite sequencing was conducted based on the post-bisulfite adaptor tagging (PBAT) strategy [34] using a modified protocol described recently (tPBAT protocol version 1.0) [35]. Genomic DNA spiked with unmethylated lambda DNA was bisulfite-treated. Random priming was performed using the DNA as a template. After purification of the product DNA with solid-phase reversible immobilization (SPRI) using AMPure XP (Beckman Coulter, Brea, CA, USA), the DNA was subjected to adaptor tagging with TACS-ligation [35]. The library structure was completed with two rounds of primer extension using universal and indexing primers. The library was purified with SPRI again before the molar concentration of the library was determined using a real-time PCR-based method. Libraries prepared from 24 different samples were tagged with different index sequences and pooled to serve for one lane of paired-end sequencing with 2 × 150 cycles on HiSeq X Ten at Macrogen Japan Inc. (Kyoto, Japan). The reads were mapped on a reference genome comprised of *E*. *coli* ER2796 (GenBank accession no. CP009644.1) and lambda DNA (GenBank accession no. J02459) using BMap [35]. From the methylation level of the lambda DNA, the bisulfite conversion efficiency was calculated to be 99% for each sample. The basic metrics of sequencing data are shown in **S1 Table**.

Cytosines mapped with more than 20 reads and detected with equal to or more than 80% of methylated reads were assumed as methylated. Methylated cytosines and their flanking 10 bp nucleotide sequences were extracted and analyzed with WebLogo 3.6.0 [36] for detection of the recognition sequence of M.BatI.

### Digestion of 60 bp dsDNA

Each strand of oligo DNA was synthesized by IDT (Coralville, IA, USA) with their sequences listed in **S2 Table**. For the formation of dsDNA, 100 μM of both strands of oligo DNA were mixed in 20 mM Tris-HCl pH8.0 and heated at 96°C for 5 min, followed by gradual cooling at room temperature for an hour.

As M.BatI showed severe toxicity in *E*. *coli* cells, M.BatI protein was prepared by *in vitro* translation using PURExpress *in vitro* protein synthesis kit (New England Biolabs, Ipswich, MA, USA). A PCR fragment with T7 promoter and *batIM* gene was used as a template. Since the product of *in vitro* translation of M.BatI was insoluble, the pellet fraction was dissolved in 100 μl of solubilization buffer (6 M Urea, 50 mM NaCl, 20 mM Tris HCl pH 8.0), followed by dialysis with dialysis buffer (20 mM Tris-HCl pH8.0, 50 mM sodium chloride) for 18 hours at 4°C.

For methylation of dsDNA by M.BatI, 400 nM of the dsDNA samples were treated with 280 nM of M.BatI with 0.1 mM S-adenosylmethionine in reaction buffer (20 mM Tris-HCl pH8.0, 50 mM potassium acetate, 5 mM EDTA-2Na, 1 mM dithiothreitol) at 37°C for 16 h. After purification with MinElute PCR Purification kit (QIAGEN, Hilden, Germany), dsDNA samples were treated with restriction enzymes for 4 h at 37°C for HaeIII, AluI, and HpyCH4V (New England Biolabs, Ipswich, MA, USA) and at 60°C for BstUI (New England Biolabs, Ipswich, MA, USA), followed by separation with 15% polyacrylamide gel electrophoresis.

### Measurement of time-kill curves

An overnight culture of *E*. *coli* strain was diluted 1000-fold in 1 ml LB and cultured with shaking at 37°C for 3 h. Induction was started with the addition of aTc to a final concentration of 100 ng/ml or different concentration when specified, then colony forming units were measured every hour. For the uninduced control, the same volume of distilled water was added instead of aTc. Measurements were biologically triplicated.

### Computational analysis of M.BatI homologs

M.BatI homologs were searched in the non-redundant (nr) database using Blastp [37] with the amino acid sequence of M.BatI as a query. Hits with both length coverage larger than 80% and sequence identity larger than 80% were selected as homologs. To analyze the genetic context of the flanking regions of the homologs, genome sequences that possessed a M.BatI homolog were downloaded from the NCBI nucleotide database. Then, the nucleotide sequence of M.BatI homologs together with flanking 15 kb sequences were extracted. Homologs were omitted from further analysis when either one or both flanking sequences were not available for 15 kb. The extracted nucleotide sequences were clustered by MeShClust [38] with 60% sequence identity as a threshold. For detection of prophages without M.BatI, 15 kb nucleotide sequences of flanking regions of *batIM* were used as a query of Blastn [37] against the nr database and top hits which were not of *B*. *anthracis* were selected.

## Results

### M.BatI produces two sequences with different methylation patterns: One fully-methylated and one hemi-methylated

**Fig 1:**
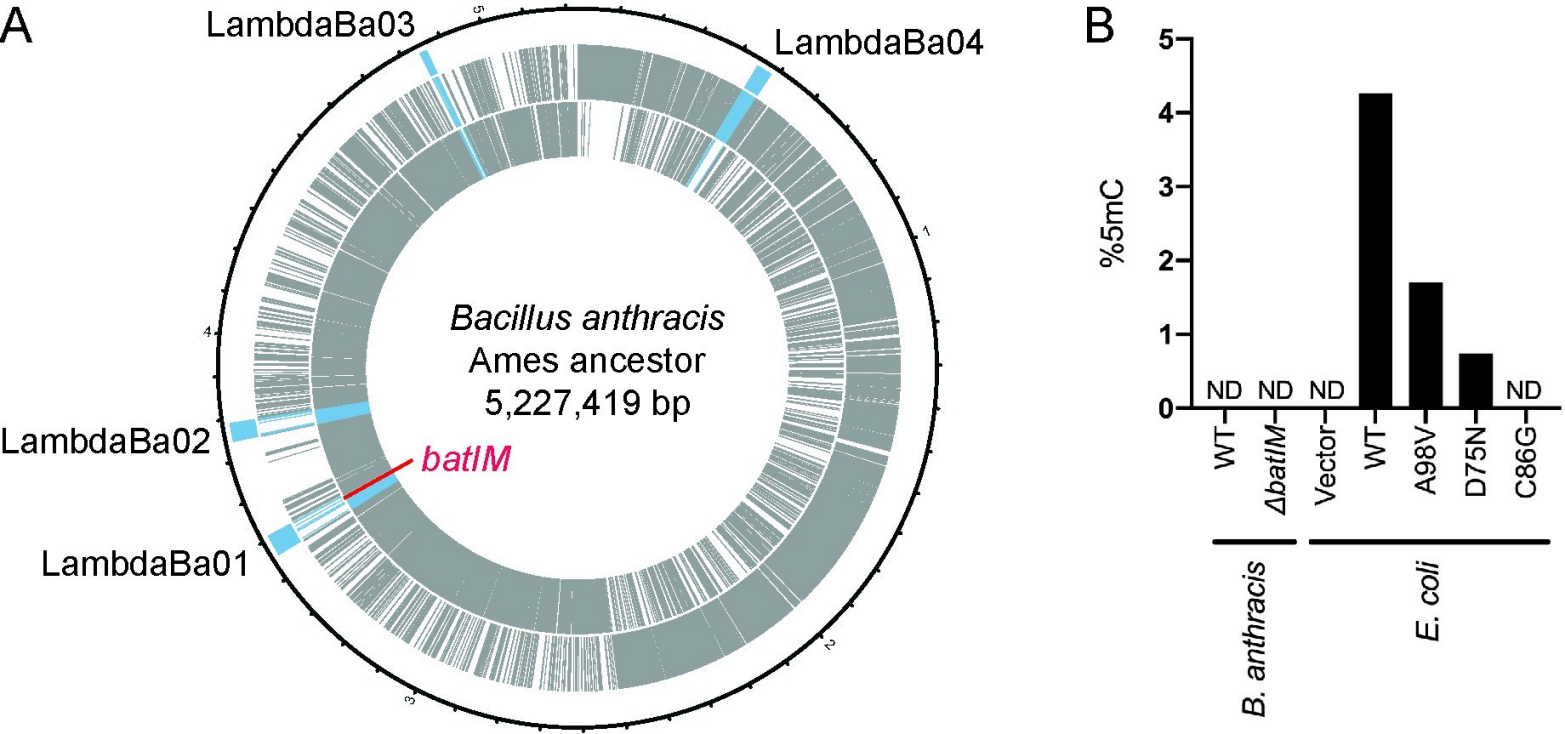
Position of *batIM* and its activity *in vivo*. (A) A map of the chromosome of *B*. *anthracis* Ames ancestor. The outermost blue bars represent the region of prophages. Gray bars represent the position of coding sequences: outer, coding sequences on the leading strand; inner, coding sequences on the lagging strand. Prophage genes were colored blue and the *batIM* gene was colored red. (B) Measurement of 5mC using UPLC-MS/MS for genomic DNA isolated from *B*. *anthracis* strains and M.BatI induced *E*.*coli* strains. ND, not detected.

The chromosomal DNA of *B*. *anthracis* possesses four prophage regions [39,40]. One of them, LambdaBa01, included a gene annotated as GBAA_RS18520 in *B*. *anthracis* Ames ancestor strain (Genbank accession No. NC_007530), which codes MTase. The gene, named *batIM*, is present in the genome without the paired gene of a restriction enzyme, thus *batIM* was highly likely to be an orphan MTase gene (**Fig 1A**). The amino acid sequence predicted from the nucleotide sequence of the *batIM* gene included conserved motifs of DNA methyltransferase that produce 5mC rather than 6mA or 4mC [41–43] (**S2 Fig**). Although most of the conserved motifs of 5mC MTase were well conserved in M.BatI, it had unusual motif I (CxGxxG) compared to that of the majority (FxGxG) (**S2 Fig**). Together with the 20–30% smaller gene size compared to typical 5mC MTases, *batIM* could form a new subclass of 5mC MTase.

Although the open reading frame seemed to be intact, M.BatI was supposed to be inactive in *B*. *anthracis in vivo* because *B*. *anthracis* was reported to express a restriction enzyme that digests cytosine-methylated DNA [24] and because prophage genes were often repressed when integrated in the host genome [44]. For validation of the methylation activity of M.BatI in *B*. *anthracis in vivo*, we extracted the genomic DNA of *B*. *anthracis*, digested into single nucleotides, and measured the amount of dC and 5mdC using UPLC-MS/MS. Neither wildtype nor *batIM* knockout strain of *B*. *anthracis* was detected with 5mdC, indicating that M.BatI was not active and no cytosine methylation occured on the genomic DNA of *B*. *anthracis in vivo* (**Fig 1B**).

To further analyze the activity of M.BatI, we cloned the *batIM* gene in *E*. *coli*. Wildtype M.BatI inducible plasmid was constructed and transformed in the *E*. *coli* ER2796 strain (**S1 Fig** and **S1 File**). Genomic DNA extracted from cells induced with M.BatI production was digested into single nucleotides and 5mdC levels were determined by UPLC-MS/MS. This analysis confirmed that the methylation product of M.BatI was 5mC (**Fig 1B**) and that M.BatI can be expressed in *E*. *coli* with its DNA methylation activity.

**Fig 2:**
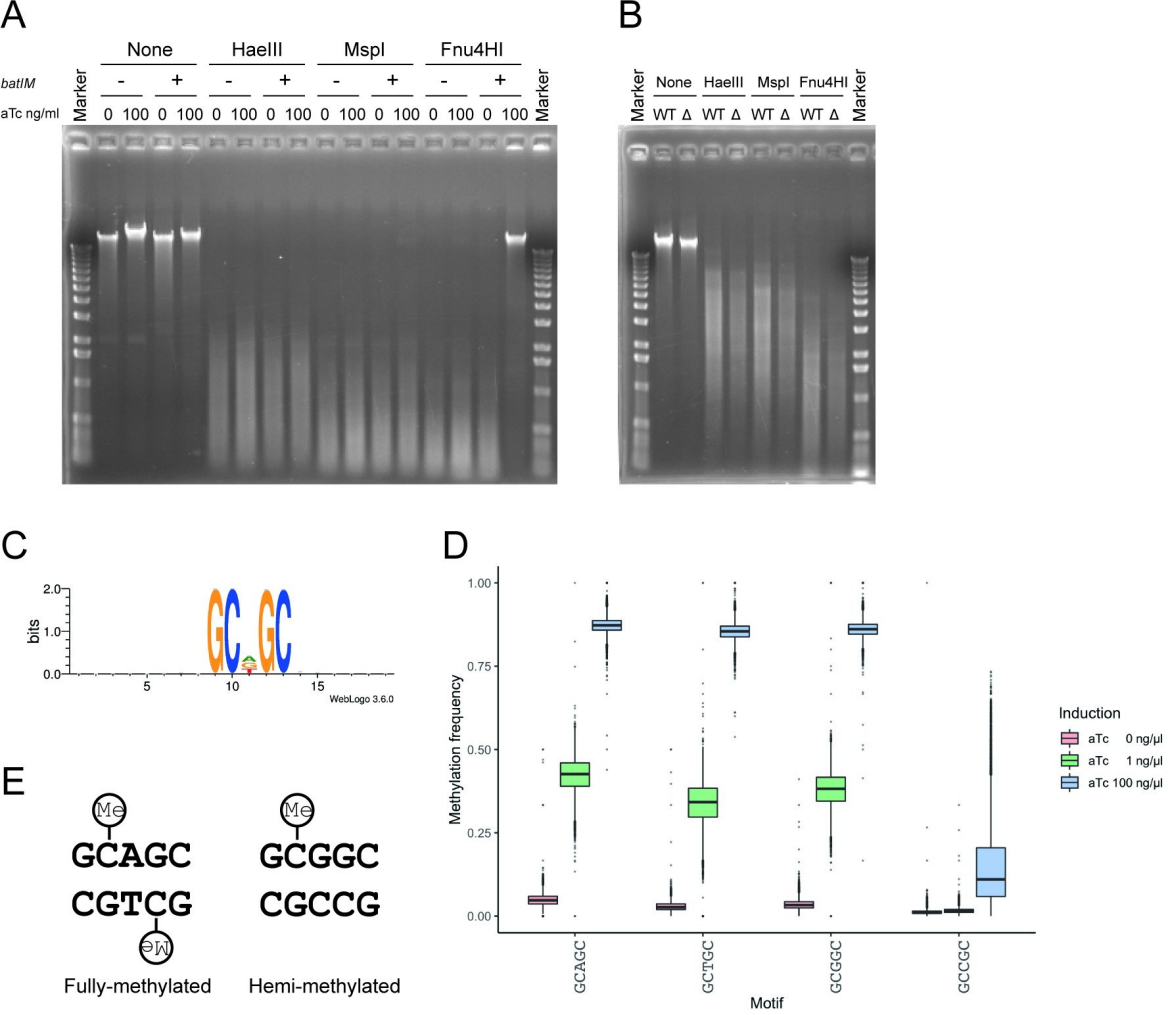
M.BatI fully-methylates 5’-GCWGC-3’ and hemi-methylates 5’-GCSGC-3’. (A) Digestion of genomic DNA of *E*. *coli* after inducing M.BatI production. Genomic DNA isolated from BYF823 (*batIM*-) or BYF822 (*batIM*+) induced with aTc 0 or 100 ng/ml was treated with specific restriction enzymes or untreated as controls. (B) Digestion of genomic DNA of wildtype (WT) and *batIM* knockout (Δ) strains of *B*. *anthracis*. (C) Sequence logo of sites around cytosines detected, by bisulfite sequencing, to be highly methylated. (D) Boxplots of methylation frequency of each sequence included in 5ʹ-GCNGC-3ʹ. (E) Fully-methylated and hemi-methylated motifs produced by M.BatI. Methylated cytosines were depicted with “Me” in a circle.

To deduce the target sequence of M.BatI for methylation activity, modified genomic DNA was challenged by restriction enzymes. We chose HaeIII, MspI, and Fnu4HI (which digests 5ʹ-GGCC-3ʹ, 5ʹ-CCGG-3ʹ, and 5ʹ-GCNGC-3ʹ, respectively) because MTases previously found in other *Bacillus* phages were analyzed by these restriction enzymes [45]. Genomic DNA isolated from cultures of *E*. *coli* after an hour of induction of M.BatI showed protection only from Fnu4HI digestion, suggesting that M.BatI at least methylates a sequence included in 5ʹ-GCNGC-3ʹ (**Fig 2A**). Consistent with UPLC-MS/MS analysis, such protection was not observed for genomic DNA isolated from *B*. *anthracis* (**Fig 2B**).

To determine the target sequence for methylation in more detail, the same genomic DNA samples were subjected to bisulfite sequencing. When M.BatI WT was fully induced, 2.3% (53,364/2,273,399) of cytosines throughout the genome were detected with more than 80% of methylation frequency. Motif search with the flanking sequences of methylated cytosines revealed that the first cytosine of the motif 5ʹ-GCDGC-3ʹ was methylated (**Fig 2C**). D is the degenerate base for A, T, and G, therefore the result suggested that the first cytosines were methylated for 5ʹ-GCAGC-3ʹ, 5ʹ-GCTGC-3ʹ, and 5ʹ-GCGGC-3ʹ, but not for 5ʹ-GCCGC-3ʹ. Consistently, the fraction of methylation signals at each motif included in 5ʹ-GCNGC-3ʹ showed a significantly lower distribution of the signal only for 5ʹ-GCCGC-3ʹ (**Fig 2D**). These results suggested that methylation by M.BatI results in full-methylation and hemi-methylation of 5ʹ-GCWGC-3ʹ and 5ʹ-GCSGC-3ʹ, respectively (**Fig 2E**). This finding was consistent with the results of the Fnu4HI digestion of genomic DNA (**Fig 2A**) because hemi-methylation of the motif was reported to be enough for protection from digestion by Fnu4HI [46].

**Fig 3:**
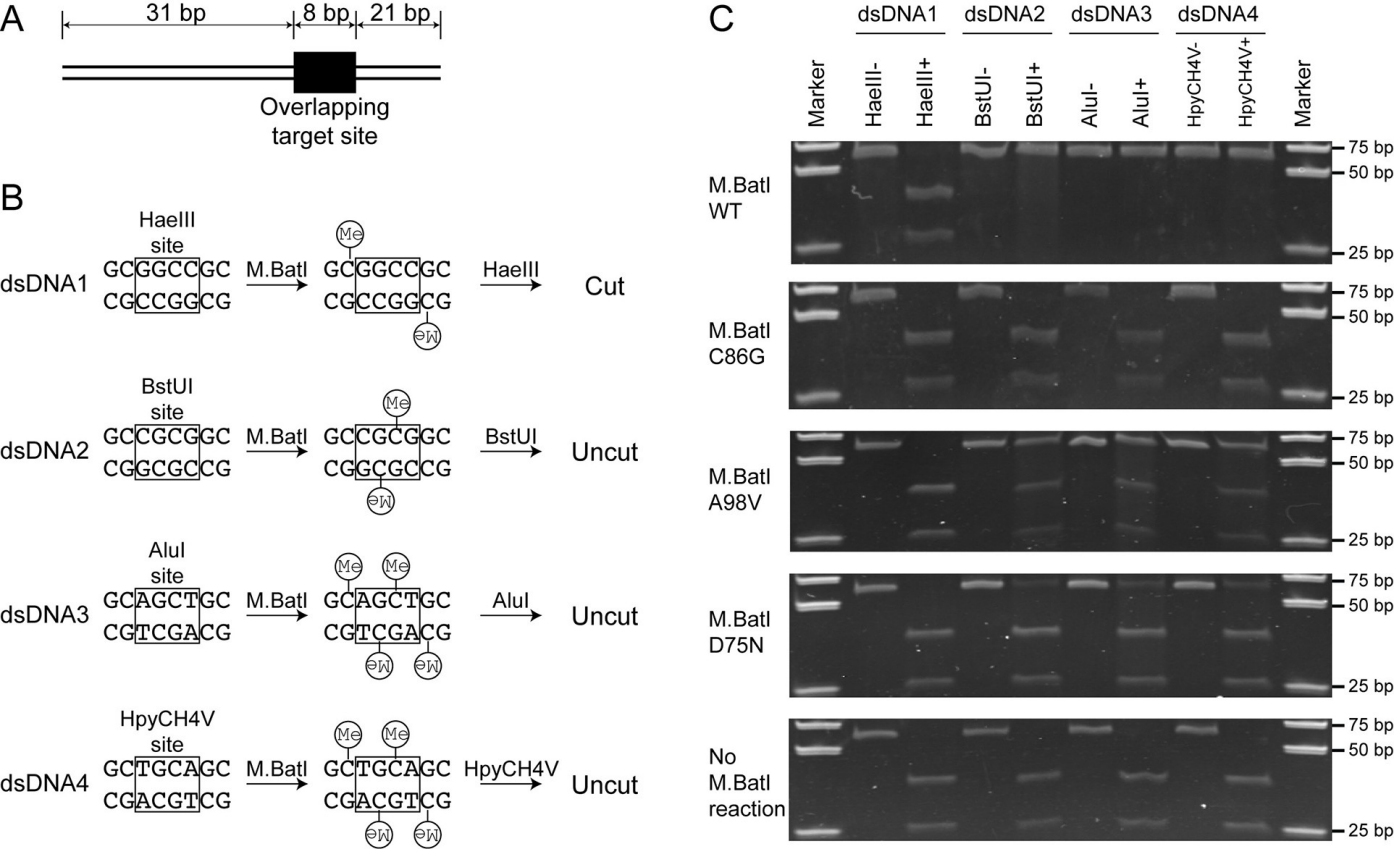
Target sequence confirmation of M.BatI by digestion of M.BatI methylated dsDNA. (A) Structure of 60 bp dsDNA. The site with overlapping sequences was positioned at one-third from the right end. (B) Design of overlapping sites in each dsDNA and the expected outcomes by methylation and digestion reactions. Each site included a target site of another restriction enzyme (squares). Expected positions of methylation were depicted with “Me” in a circle. (C) Digestion tests of dsDNA. Each dsDNA was first treated with M.BatI and digested with one of the four restriction enzymes.

To further confirm the target sequence of M.BatI, methylation activity for four target sequences included in 5’-GCNGC-3’ was tested with methylation and digestion of short dsDNA oligos (**Fig 3A and 3B**). The dsDNA oligoduplexes were synthesized to contain two overlapping subsites of the M.BatI target sequence. The M.BatI target sites were placed to form the recognition site of a restriction endonuclease of known methylation sensitivity. In dsDNA1, the overlapping M.BatI sites created a HaeIII recognition site (5ʹ-GGCC-3ʹ) [18]. If M.BatI does not methylate the first cytosine of 5ʹ-GCCGC-3ʹ, the HaeIII site will be left unmethylated after the treatment with M.BatI, and will be digested by HaeIII. In dsDNA2, the overlapping M.BatI sites created a BstUI site (5ʹ-CGCG-3ʹ) [18]. If M.BatI methylates the first cytosine of 5’-GCGGC-3’, the BstUI site will get fully-methylated by M.BatI, and will be protected from BstUI digestion. The duplexes dsDNA3 and dsDNA4 were designed to analyze the methylation of the 5’-GCTGC-3’ and 5’-GCAGC-3’ subsites (**Fig 3B**). By challenging each dsDNA with corresponding restriction enzymes after treatment with M.BatI, methylation of four sequences included in 5ʹ-GCNGC-3ʹ was tested.

Each dsDNA was treated with M.BatI prepared by *in vitro* translation, then it was digested with the corresponding restriction enzyme. Digestion was observed only in one case: dsDNA1-HaeIII (**Fig 3C, top panel**). This result indicated that M.BatI methylate the first cytosine of 5ʹ-GCGGC-3ʹ, 5ʹ-GCAGC-3ʹ, and 5ʹ-GCTGC-3ʹ, but not of 5ʹ-GCCGC-3ʹ, consistent with the result of bisulfite sequencing above.

We concluded that the target sequence of M.BatI is the combination of full-methylation of 5ʹ-GCWGC-3ʹ and hemi-methylation of 5’-GCSGC-3’, instead of 5ʹ-GCNGC-3ʹ. To the best of our knowledge, this is the first report of a single MTase that methylates one or both strands of subsites of a degenerate recognition sequence (**Fig 2E**).

The guanine 2-amino group protruding in the minor groove was shown to mediate differentiation between A/T and G/C by DNA binding proteins [47–49]. To test whether the guanine 2-amino group has any role in the exclusion of the 5’-GCCGC-3’ sequence by M.BatI, we prepared dsDNA substrate, in which the guanine opposite to the underlined cytosine was substituted with hypoxanthine: 5’-GCCGC-3’/5’-GCIGC-3’. Hypoxanthine lacks the 2-amino group but still pairs with cytosine. The result indicated that the 2-amino group has no role determining the specificity of M.BatI (**S3 Fig**). Results of structural analysis of the major and minor groove surfaces suggest that the W2/W2’ position in the major groove might be the place where recognition (rejection) occurs, because this is the place where cytosine differs from the other three bases [48].

### M.BatI was active and toxic in *E*. *coli*

**Fig 4:**
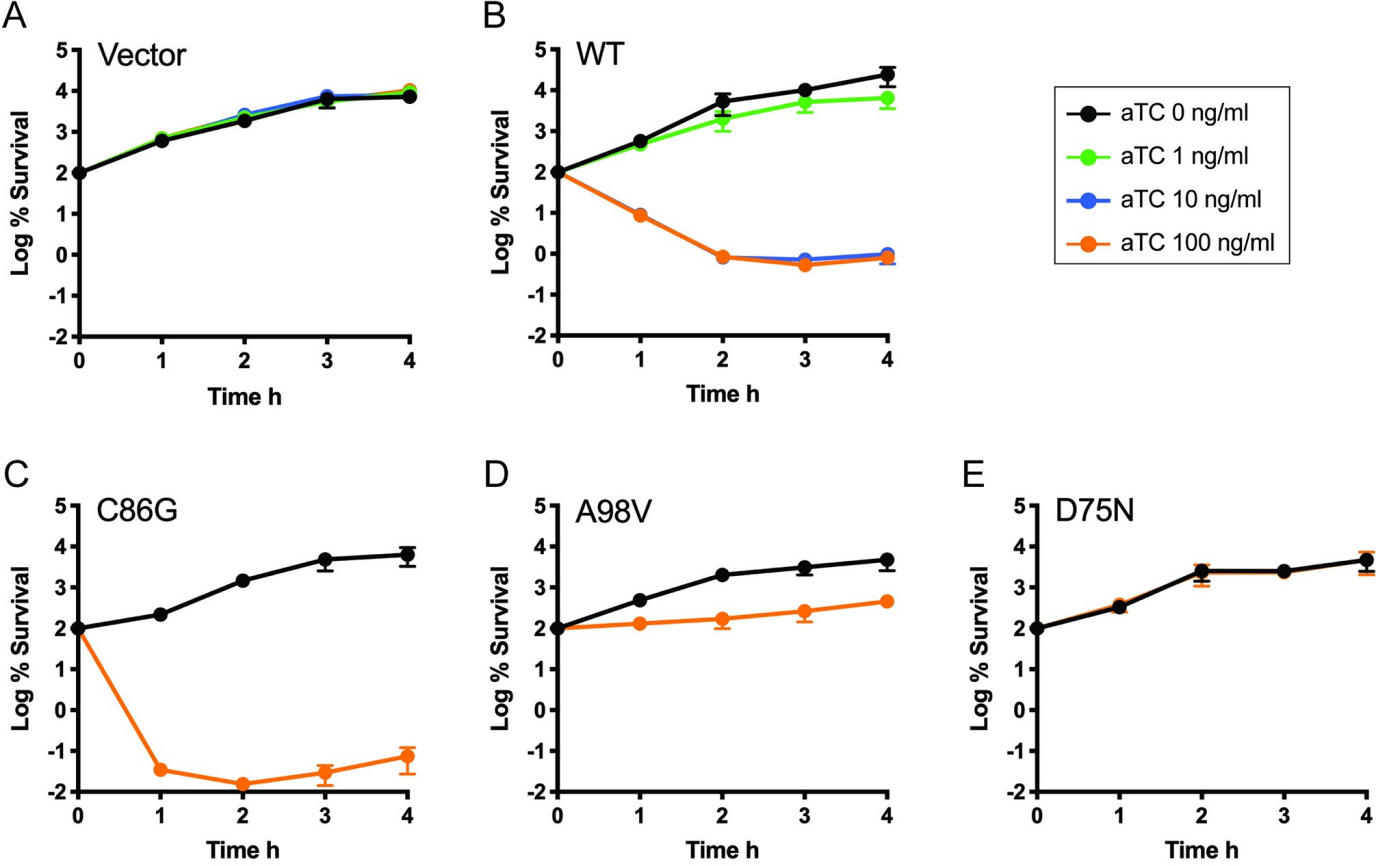
Toxicity of M.BatI variants in *E*. *coli*. The number of colony forming units was determined after induction at 0 h with aTc for *E*. *coli* strains expressing (A) no M.BatI, (B) wildtype M.BatI, (C) M.BatI C86G, (D) M.BatI A98V, and (E) M.BatI D75N. Black, no induction; Green, aTc 1 ng/ml; Blue, aTc 10 ng/ml; Orange, aTc 100 ng/ml.

**Fig 5:**
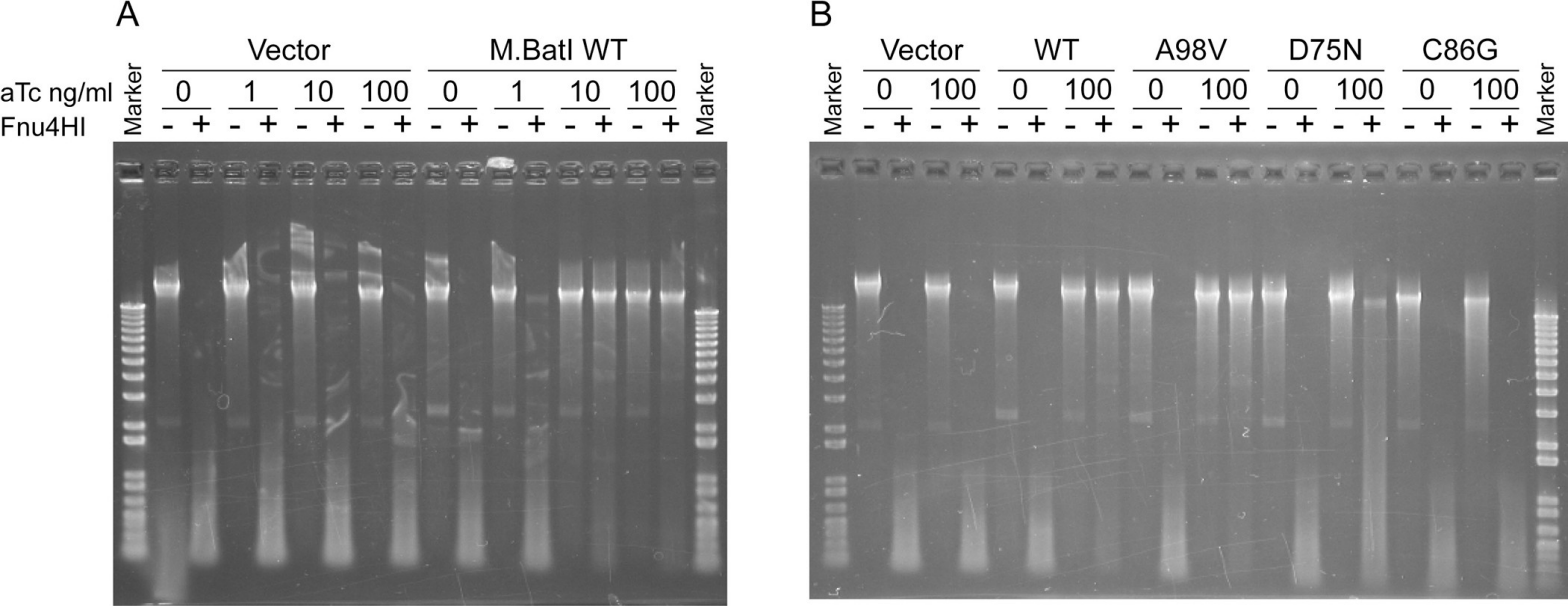
Genomic DNA modification by M.BatI with different levels of induction and mutations. (A) Digestion of genomic DNA induced with different concentrations of aTc. (B) Digestion of genomic DNA modified by M.BatI variants.

We noticed that M.BatI killed *E*. *coli* cells when it was induced above a certain level (**Fig 4A and 4B**). Analysis of *E*. *coli* genomic DNA extracted from cultures after an hour of induction at various levels showed that genomic DNA isolated from the condition with more cell death showed higher distribution of methylation signals in bisulfite sequencing and greater protection against Fnu4HI digestion (**Figs 2D and 5A**). This result motivated us to investigate whether the toxicity of M.BatI was caused by its DNA methylation activity.

We first tested if the complete abolishment of methylation activity decreased the toxicity by constructing a variant lacking DNA methylation activity by substituting the 86th cysteine residue with glycine at the activity center of M.BatI. Loss of DNA methylation activity was confirmed clearly by the lack of protection from digestion by restriction enzymes and the complete loss of 5mC signal in the measurement using UPLC-MS/MS (**Figs 1B, 3C and 5B**). The C86G variant, however, still showed toxicity comparable to WT when induced (**Fig 4C**).

We also attempted whether we can isolate variants that maintain DNA methylation activity but with lower toxicity. The plasmid expressing M.batI was transformed into *E*. *coli* ER2796 and selected with induction of M.BatI. All survived colonies were pooled and were used for preparation of plasmids. The plasmids were digested with Fnu4HI to remove variants without methylation activity, then transformed in ER2796 with a fresh background. By analyzing the final surviving transformants, we successfully isolated two M.BatI variants: A98V and D75N. A98V showed a bacteriostatic effect rather than bactericidal, while D75N showed no toxicity (**Fig 4D and 4E**). DNA methylation activity was maintained in both A98V and D75N, but the level of methylation was lower than in WT as shown by UPLC-MS/MS measurement and in the DNA digestion experiment (**Figs 1B, 3C and 5B**), suggesting that methylation activity could have correlation with the toxicity.

We concluded from these analyses that the toxic effects of M.BatI in *E*. *coli* is caused by a mechanism other than its DNA methylation activity.

### Genomic context of M.BatI homologs

**Fig 6:**
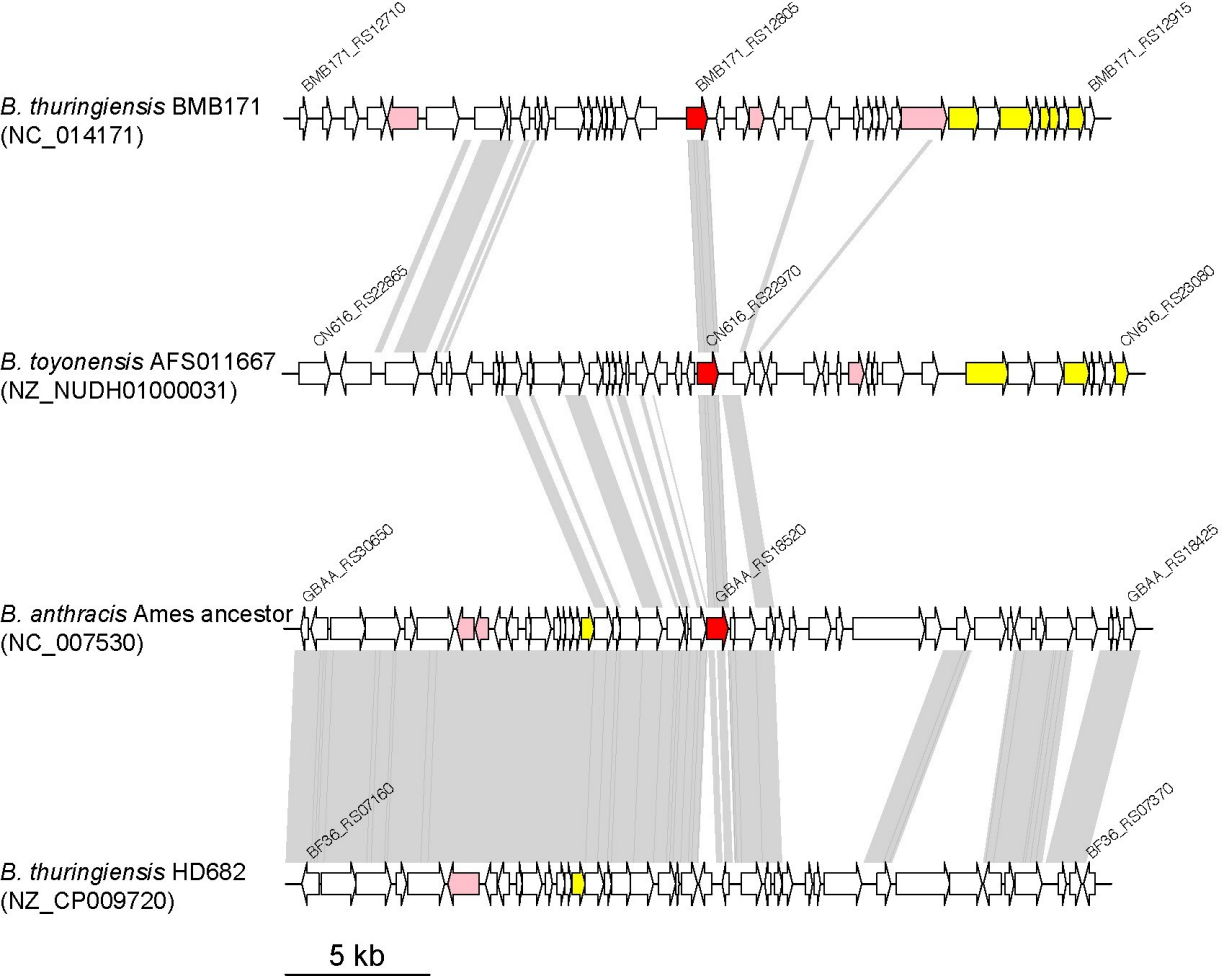
Genomic context of *batIM* and its homologs. Red, *batIM* and its homologs; pink, genes annotated as terminase, integrase, or recombinase encoding genes; yellow, annotated as head, tail, or other structural proteins encoding genes.

The chromosome sequence of *B*. *anthracis* is highly conserved, thus the *batIM* gene was present in all *B*. *anthracis* strains which were deposited with complete genome sequences in NCBI. Other than *B*. *anthracis*, homologs of *batIM* were found in other species of *B*. *cereus* group such as *Bacillus thuringiensis* (BMB171_RS12805) and *Bacillus toyonensis* (CN616_RS22970) (**Figs 6 and S2**). When sequences of *batIM* homologs and their flanking 15 kb regions were compared, sequences were clustered into three groups, suggesting that *batIM* homologs were present in several different genomic contexts. Annotation of genes in the flanking regions revealed that all homologs were found in the vicinity of genes coding phage related enzymes such as integrase, recombinase, and phage parts proteins, suggesting that all *batIM* homologs were possessed by prophages. Therefore, the homologs would have spread among some of the *B*. *cereus* group species by several different phages.

A similar prophage of *B*. *anthracis* was, however, found in other species with inactivated *batIM* homologs. For example, *Bacillus thuringiensis* HD682 possessed a prophage similar to that in *B*. *anthracis* (**Fig 6**, bottom), but the *batIM* homolog was truncated. Therefore, *batIM* homologs might be not always required for the life cycle of the prophage and also repressed and inactivated in species other than *B*. *anthracis*.

## Discussion

We characterized M.BatI, the orphan 5mC MTase of *B*. *anthracis*. It showed no methylation activity in *B*. *anthracis in vivo* but showed activity and even toxicity when expressed in *E*. *coli*. Bisulfite sequencing elucidated that the enzyme produces two sequences with different methylation patterns: 5’-GCWGC-3’ with full-methylation and 5’-GCSGC-3’ with hemi-methylation. Its homologs were found on different prophage like regions in other species of the *B*. *cereus* group, suggesting the spread of the gene by several different phages.

Among MTases, 6mA and 4mC MTase have common conserved motifs while the 5mC MTase consists of different conserved motifs [41,43]. Although many mammalian species mainly possess 5mC MTase and DNA modification patterns were analyzed mainly by bisulfite sequencing, SMRT sequencing by PacBio sequencers were mainly used for epigenetic analyses of bacterial species probably because of its higher sensitivity for detection of 6mA and higher prevalence of 6mA MTase in bacteria. The SMRT sequencing, however, has a much lower sensitivity for 5mC unless a sequencing library was prepared with additional treatment [50]. Therefore, many of the bacterial methylome analyses by SMRT sequencing failed to detect motifs of 5mC MTases. Analysis of bacterial 5mC MTase by bisulfite sequencing was conducted only in a few studies [51–55]; thus, accumulation speed of target sequences of bacterial 5mC MTase was slower than that of bacterial 6mA MTase. The discovery of the unique target sepcificity of M.BatI implies that the diversity of target sequences of bacterial cytosine MTases have yet to be fully elucidated and more analysis with genome-wide methods should be conducted for bacterial 5mC MTase.

Bacterial MTases usually have a single recognition sequence, which is either fully- or hemi-methylated by the enzyme. M.BatI acts differently on subsites of its degenerate recognition sequence: it methylates one subsite on both strands and another subsite only on one strand (**Fig 2E**). MTases with multiple target sequences were already reported for MTases of phages of *Bacillus* species [45,56,57]. In the coding sequences, they contained multiple target recognition domains positioned in tandem and resulted in multiple recognition sequences [58,59]. This was, however, not the case for M.BatI because M.BatI had an even shorter length of target recognition domain compared to a single target recognition domain of MTases (**S2 Fig**). In addition, all the multiple recognition sequences of the *Bacillus* phage MTases produced fully-methylated sites. Therefore, although *batIM* was present on the prophage region of *B*. *anthracis*, the mechanism for possessing unique target sequence and methylation patterns seems to be different from those of other *Bacillus* phage MTases with multiple target sequences. Some residues in the target recognition domain of M.BatI might interrupt binding or methylation reaction against 5ʹ-GCCGC-3ʹ specifically, but the detail of the mechanism remains unknown.

According to the REBASE [18], the largest database of restriction and modification enzymes, a target sequence of MTases close to that of M.BatI, which could be represented as 5ʹ-GCDGC-3ʹ using degenerate base, was observed in a few results of PacBio sequencing of *Bacillus* species FDAARGOS_235, *Bacillus licheniformis* SCDB 14, and *Streptococcus oralis* FDAARGOS_367 (**S3 Table**). Although all the similar motifs were reported to include 4mC as a methylation product, their methylation frequency was always less than 31%. These observations suggest the possibility of miscalls of 5mC because PacBio usually detects 4mC with high sensitivity comparable to 6mA. *Bacillus* species FDAARGOS_235 possessed a homolog of M.BatI, but the other two did not, thus the recognition motif including both fully-methylated and hemi-methylated might be found not only with M.BatI but also with other MTases.

Because DNA methylation activity of bacterial MTases was known to affect the phenotypes of cells, we first expected that the toxicity of M.BatI in *E*. *coli* cells could be due to DNA methylation activity, but our observations showed that the toxicity was caused possibly by other factors. One possible explanation is its tight binding to the genomic DNA on target sequences, which was previously suggested for toxic variants of other 5mC MTases such as M. EcoRII and M. HhaI [60–62]. In these cases, substitution of the cysteine to glycine at the catalytic center led to toxicity against *E*. *coli* cells. The two mutations we found in M.BatI, A98V and D75N, might affect the binding activity and resulted in less toxicity, which also resulted in the decrease of DNA methylation activity. Unusual tight binding of MTases to the DNA substrate was suggested as a source of the toxicity, but the mechanism was not yet understood in detail [41] and biochemical analyses are required to validate this hypothesis.

In conclusion, we characterized the specificity of a bacterial cytosine MTase, M.BatI, with a unique activity that results in one fully-methylated and one hemi-methylated sequence. To our knowledge, this is the first discovery of bacterial cytosine MTase that produces both fully-methylated and hemi-methylated sites. More investigation of the recognition sequences of bacterial cytosine MTases by genome-wide analysis such as bisulfite sequencing would lead to the discovery of new recognition sequences and possibly to an expansion of recognition sequence diversity of MTases.

## Supporting information

S1 Fig Map of pZE31-tetR-batIM. (PDF)

S2 Fig Alignment of M.BatI and its homologs together with other representative cytosine methyltransferase. Conserved motifs of cytosine methyltransferase were colored. The region corresponding to target recognition domain were indicated with red line above the alignment. In M.BatI sequence, letters of residues of variants we constructed were colored in red.
(PDF)

S3 Fig DNA bond structure at the minor groove does not affect M.BatI activity. (A) Structure of 60 bp of dsDNA, same as Fig 3A. (B) Sequences of overlapping target sites in each dsDNA. Third guanine was substituted with hypoxanthine, denoted as “I”. (C) Digestion tests of dsDNA.
(PDF)

S1 Table Summary of bisulfite sequencing. (XLSX)

S2 Table Oligo DNA sequences. (XLSX)

S3 Table PacBio results in REBASE with motifs close to M.BatI. (XLSX)

S1 File Annotation and sequence of pZE31-tetR-batIM in Genbank format. (PDF)

S1 Raw images (PDF)
