# Supplementary figures and images for: A GCDGC-specific DNA (cytosine-5) methyltransferase that methylates the GCWGC sequence on both strands and the GCSGC sequence on one strand
Source: PLoS One. 2022 Mar 21;17(3):e0265225. doi: 10.1371/journal.pone.0265225 (PMC8936443; doi:10.1371/journal.pone.0265225)

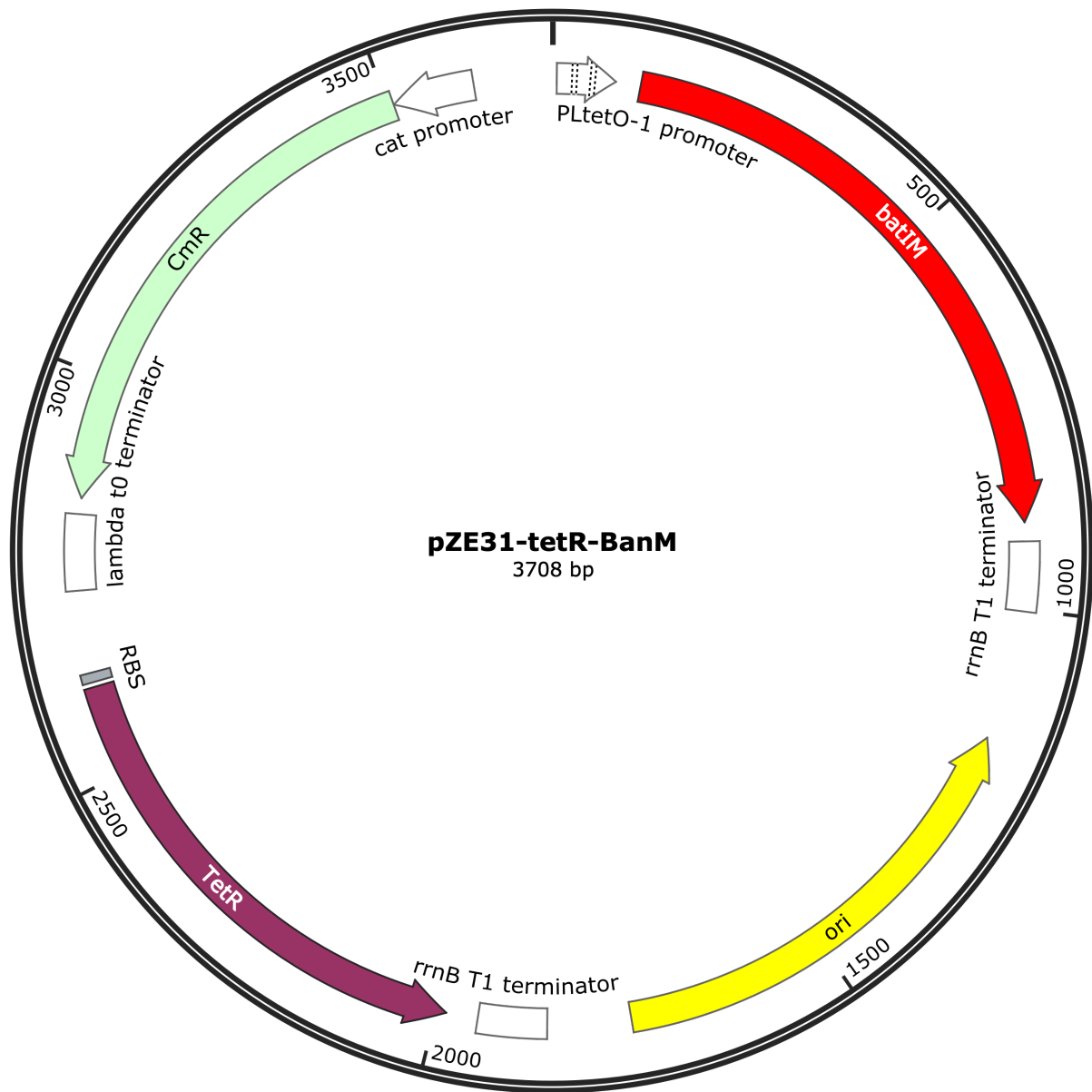

**S1 Fig. Map of pZE31-*tetR*-*batIM*.**

Supplement: S1 Fig — (PDF) [file pone.0265225.s001.pdf]
