# Supplementary material for: A GCDGC-specific DNA (cytosine-5) methyltransferase that methylates the GCWGC sequence on both strands and the GCSGC sequence on one strand
Source: PLoS One. 2022 Mar 21;17(3):e0265225. doi: 10.1371/journal.pone.0265225 (PMC8936443; doi:10.1371/journal.pone.0265225)
Supplement: S2 Fig — Conserved motifs of cytosine methyltransferase were colored. The region corresponding to target recognition domain were indicated with red line above the alignment. In M.BatI sequence, letters of residues of variants we constructed were colored in red. (PDF) [file pone.0265225.s002.pdf]

```
                                            Motif I
                     1
GBAA_RS18520(M.BatI) ---MYRSKES GLKMLDLCSG IAGISMAADW A--GI-DTAA FCEIE----- EFNQKVLRKN
BMB171_RS12805       ---------- -MKMLDLCSG IAGISMAADW A--GI-NTAA FCEIE----- EFNQKVLRKN
CN616_RS22970        ---------- -MKMLDLCSG IAGVSMAADW A--GI-ETAA FCEIE----- EFNQKVLRKN
HhaIM                MIEIKDKQLT GLRFIDLFAG LGGFRLALES C--GA-ECVY SNEWD----- KYAQEVYEMN
Sau3AIM              --------MN KIKVVELFAG VGGFRLGLEN TKNGIFDITW ANQWEPSRKI QHAFDCYSKR
                                                      Motif IV
                    61                       D75        C86           A98
GBAA_RS18520(M.BatI) YP-NIPIFPD LYKLMKQSLI DGGVDVDSIG VISAGYPCQG ESLV---GKR RGAEDER-WL
BMB171_RS12805       YP-NIPIFPD LYKLTKQSLI DGGVDVDSIG VISAGYPCQG ESMA---GKR KGAEDER-WL
CN616_RS22970        YP-NIPIFPD LYKLTKQSLI DGGVDVDAIG VISAGYPCQG ESLV---GKR RGAEDER-WL
HhaIM                F--GEKPEGD ITQVNEKTIP DH--D----- ILCAGFPCQA FSIS---GKQ KGFEDSRGTL
Sau3AIM              FKNGIHSNKD IAQVSDEEMA NTEAD----- MIVGGFPCQD YSVARSLNGE LGIQGKKGVL

                   121            Motif VI
GBAA_RS18520(M.BatI) WPEVFRLIKE LRPTWFVGEN V-------AG HVTMGLDTVL SDLEEENYST RTFVFPAVSV
BMB171_RS12805       WPEVFRLIRE LRPTWFVGEN V-------AG HVTMGLDTVL SDLEEENYST RTFVLPAVSV
CN616_RS22970        WPEVFRLIRE LRPTWFVGEN V-------AG HVTMGLDTVL SDLEEENYST RTFVLPAVSV
HhaIM                FFDIARIVRE KKPKVVFMEN VKNF---ASH DNGNTLEVVK NTMNELDYSF HAKVLNALDY
Sau3AIM              FWQIIRYIQN TFPKYLLLEN VDRLLKSPSS QRGRDFAVML STLNELGYNV EWRVINAADY

                   181 Motif VIII
GBAA_RS18520(M.BatI) GAPHQRYRTF IVGHSNDKS- ---------- KLQTDPRVVP FRSKWETWEN TTGINRGTLS
BMB171_RS12805       GAPHQRYRTF IVGHSNDKP- ---------- KLQANKKIIP FRSKRQPWKT TSGIDRGALS
CN616_RS22970        GAPHQRYRTF IIGHSNDKS- ---------- KLQADPRVVP FRSKREARKN TTRINRGTLS
HhaIM                GIPQKRERIY MICFRNDLN- ---------- ---IQNFQFP KPFELNTFVK DLLLPDSEVE
Sau3AIM              GNAQRRRRVF IFGYKQDLNY SKAMEESPLD KIIYHNGLFA EAFPIEDYAN KNRVNRTHIT

                   241                         Target recognition domain
GBAA_RS18520(M.BatI) ---------- ---------- ---------- ---------- ---------- ----------
BMB171_RS12805       ---------- ---------- ---------- ---------- ---------- ----------
CN616_RS22970        ---------- ---------- ---------- ---------- ---------- ----------
HhaIM                HLVIDRKDL- ---------VM TNQEIE--QT TP---KTVRL G--------- ----------
Sau3AIM              HDIVDISDNF SFQFYNSGIM KNGEILTIDT IPKYEKSVTL GEIIESNVDD GFSLNQDQID

                   301
GBAA_RS18520(M.BatI) ---------- ---------G TYWEEN---- ---------- ---------- ----------
BMB171_RS12805       ---------- ---------G TYWEEN---- ---------- ---------- ----------
CN616_RS22970        ---------- ---------R IYWEEN---- ---------- ---------- ----------
HhaIM                ---------- IVGKGGQGER IYSTRG---- ------IAIT LSAYGGGIFA KTGGYLVNGK
Sau3AIM              KFKYLRGPKK IKRTTKDGHE YYFSEGGMSE TDSLELPART MLTSESSINR STHFLNVDGV

                   361  (Motif IX)                    Motif X
GBAA_RS18520(M.BatI) ----KPAIC- ---GMDDGTA TRLD-EDRLR FLGNAVVPQQ IYPIFEAIAK IEGLL-----
BMB171_RS12805       ----KPAVC- ---GMDDGTA ARLD-EDRLR FLGNAVVPQQ IYPIFEAIAK IEGLL-----
CN616_RS22970        ----QPAIC- ---GMDDGTA TRLD-EDRLR FLGNAVVPQQ IYPIFEAIAK IEGLL-----
HhaIM                TRKLHPRECA RVMGYPDSYK VHPSTSQAYK QFGNSVVINV LQYIAYNIGS SLNFK-----
Sau3AIM              YRTLTPIEAE RLNGFPDNWT EGMPIKMRYF CMGNALVVPL ITRIGNQIEK IDSITNDEFS

                   421
GBAA_RS18520(M.BatI) -----
BMB171_RS12805       -----
CN616_RS22970        -----
HhaIM                ---PY
Sau3AIM              QLRLF
```

S2 Fig. Alignment of M.BatI and its homologs together with other representative cytosine methyltransferase. Conserved motifs of cytosine methyltransferase were colored. The region corresponding to target recognition domain were indicated with red line above the alignment. In M.BatI sequence, letters of residues of variants we constructed were colored in red.
