# Supplementary material for: A GCDGC-specific DNA (cytosine-5) methyltransferase that methylates the GCWGC sequence on both strands and the GCSGC sequence on one strand
Source: PLoS One. 2022 Mar 21;17(3):e0265225. doi: 10.1371/journal.pone.0265225 (PMC8936443; doi:10.1371/journal.pone.0265225)
Supplement: S3 Fig — (A) Structure of 60 bp of dsDNA, same as Fig 3A. (B) Sequences of overlapping target sites in each dsDNA. Third guanine was substituted with hypoxanthine, denoted as “I”. (C) Digestion tests of dsDNA. (PDF) [file pone.0265225.s003.pdf]

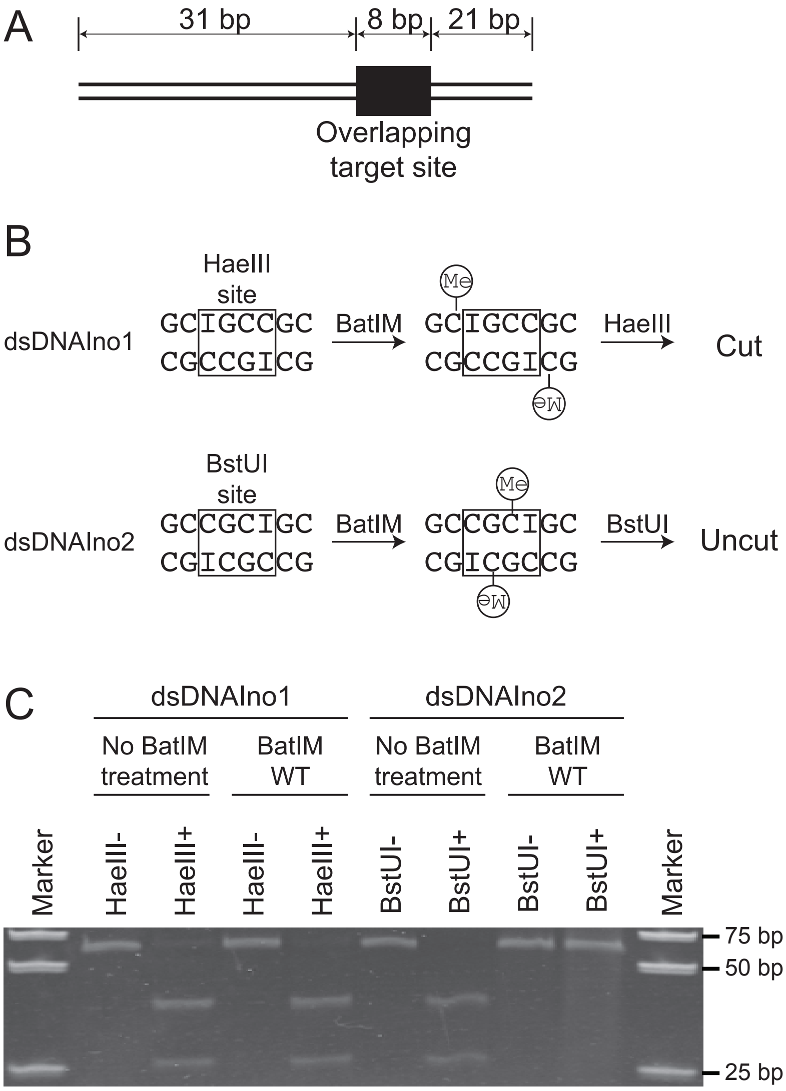

S3 Fig. DNA bond structure at the minor groove does not affect M.BatI activity. (A) Structure of 60 bp of dsDNA, same as Fig 3A. (B) Sequences of overlapping target sites in each dsDNA. Third guanine was substituted with hypoxanthine, denoted as "I". (C) Digestion tests of dsDNA.
