# Supplementary material for: A GCDGC-specific DNA (cytosine-5) methyltransferase that methylates the GCWGC sequence on both strands and the GCSGC sequence on one strand
Source: PLoS One. 2022 Mar 21;17(3):e0265225. doi: 10.1371/journal.pone.0265225 (PMC8936443; doi:10.1371/journal.pone.0265225)
Supplement: S1 File — (PDF) [file pone.0265225.s007.pdf]

Supporting Information. Annotation and sequence of pZE31-*tetR-batIM* in Genbank format.

```
LOCUS       Exported                3708 bp ds-DNA     circular SYN 21-JAN-2022
DEFINITION  synthetic circular DNA
ACCESSION   .
VERSION     .
KEYWORDS    .
SOURCE      synthetic DNA construct
  ORGANISM  synthetic DNA construct
REFERENCE   1  (bases 1 to 3708)
  AUTHORS   CZC, Hokkaido Universirty
  TITLE     Direct Submission
  JOURNAL   Exported Friday, Jan 21, 2022 from SnapGene 5.0.8
            https://www.snapgene.com
FEATURES             Location/Qualifiers
     source          1..3708
                     /organism="synthetic DNA construct"
                     /mol_type="other DNA"
     promoter        7..80
                     /label=PLtetO-1 promoter
                     /note="modified phage lambda PL promoter with tet operator
                     sites (Lutz and Bujard, 1997)"
     protein_bind    7..25
                     /gene="tetO"
                     /label=tet operator
                     /bound_moiety="tetracycline repressor TetR"
                     /note="bacterial operator O2 for the tetR and tetA genes"
     protein_bind    32..50
                     /gene="tetO"
                     /label=tet operator
                     /bound_moiety="tetracycline repressor TetR"
                     /note="bacterial operator O2 for the tetR and tetA genes"
     CDS             112..891
                     /codon_start=1
                     /locus_tag="AW20_RS24270"
                     /old_locus_tag="AW20_4689"
                     /product="DNA methylase"
                     /label=batIM
                     /note="Derived by automated computational analysis using
                     gene prediction method: Protein Homology."
                     /inference="COORDINATES:same species:RefSeq"
                     /protein_id="WP_000286272.1"
                     /translation="MYRSKESGLKMLDLCSGIAGISMAADWAGIDTAAFCEIEEFNQKV
                     LRKNYPNIPIFPDLYKLMKQSLIDGGVDVDSIGVISAGYPCQGESLVGKRRGAEDERWL
                     WPEVFRLIKELRPTWFVGENVAGHVTMGLDTVLSDLEEENYSTRTFVFPAVSVGAPHQR
                     YRTFIVGHSNDKSKLQTDPRVVPFRSKWETWENTTGINRGTLSGTYWEENKPAICGMDD
                     GTATRLDEDRLRFLGNAVVPQQIYPIFEAIAKIEGLL"
     terminator      916..1002
                     /gene="Escherichia coli rrnB"
                     /label=rrnB T1 terminator
                     /note="transcription terminator T1 from the E. coli rrnB
                     gene"
     rep_origin      complement(1167..1755)
                     /direction=LEFT
                     /label=ori
                     /note="high-copy-number ColE1/pMB1/pBR322/pUC origin of
                     replication"
     terminator      1861..1947
                     /gene="Escherichia coli rrnB"
                     /label=rrnB T1 terminator
```

```
                     /note="transcription terminator T1 from the E. coli rrnB
                     gene"
     CDS             complement(1988..2611)
                     /codon_start=1
                     /gene="tetR from transposon Tn10"
                     /product="tetracycline repressor TetR"
                     /label=TetR
                     /note="TetR binds to the tetracycline operator tetO to
                     inhibit transcription. This inhibition can be relieved by
                     adding tetracycline or doxycycline."
                     /translation="MSRLDKSKVINSALELLNEVGIEGLTTRKLAQKLGVEQPTLYWHV
                     KNKRALLDALAIEMLDRHHTHFCPLEGESWQDFLRNNAKSFRCALLSHRDGAKVHLGTR
                     PTEKQYETLENQLAFLCQQGFSLENALYALSAVGHFTLGCVLEDQEHQVAKEERETPTT
                     DSMPPLLRQAIELFDHQGAEPAFLFGLELIICGLEKQLKCESGS"
     RBS             2618..2629
                     /note="strong bacterial ribosome binding site (Elowitz and
                     Leibler, 2000)"
     terminator      2733..2827
                     /label=lambda t0 terminator
                     /note="transcription terminator from phage lambda"
     CDS             complement(2848..3507)
                     /codon_start=1
                     /gene="cat"
                     /product="chloramphenicol acetyltransferase"
                     /label=CmR
                     /note="confers resistance to chloramphenicol"
                     /translation="MEKKITGYTTVDISQWHRKEHFEAFQSVAQCTYNQTVQLDITAFL
                     KTVKKNKHKFYPAFIHILARLMNAHPEFRMAMKDGELVIWDSVHPCYTVFHEQTETFSS
                     LWSEYHDDFRQFLHIYSQDVACYGENLAYFPKGFIENMFFVSANPWVSFTSFDLNVANM
                     DNFFAPVFTMGKYYTQGDKVLMPLAIQVHHAVCDGFHVGRMLNELQQYCDEWQGGA"
     promoter        complement(3508..3610)
                     /label=cat promoter
                     /note="promoter of the E. coli cat gene encoding
                     chloramphenicol acetyltransferase"
ORIGIN
        1 ctcgagtccc tatcagtgat agagattgac atccctatca gtgatagaga tactgagcac
       61 atcagcagga cgcactgacc tgcaatcatc caagtttcta ccggcgaaat tatgtacaga
      121 agtaaggaat ctggactgaa aatgttagat ttgtgttcgg gaattgcagg aataagtatg
      181 gcagcggatt gggctggaat tgatacagca gccttttgtg aaatagagga gttcaatcaa
      241 aaagtactta gaaagaacta tcctaacatt cctattttcc cggatttata taaacttatg
      301 aaacaatcat taatagatgg aggtgttgac gttgattcaa ttggagttat ttcagcagga
      361 tatccctgtc agggagaaag tcttgttggg aagcgaagag gtgcagaaga cgaaaagatgg
      421 ttatggccag aagtcttccg acttattaaa gaactccggc ccacttggtt tgttggagaa
      481 aatgttgctg gacacgtcac gatgggcttg gacaccgtgc tctccgactt ggaagaagaa
      541 aactactcga caaggacgtt tgtatttccg gctgtcagtg tcggcgcgcc gcatcaaaga
      601 taccggacat ttattgttgg ccactccaac gacaagtcaa aattacaaac cgatccgaga
      661 gttgtgccct tcagaagcaa atgggaaaca tgggaaaaca ctaccgggat caatcgggga
      721 acactttccg gaacatattg ggaagaaaat aaacccgcaa tttgtggaat ggatgatggg
      781 actgccacaa gattggacga agatagattg agattcttag gaaacgcggt tgtacctcag
      841 cagatttatc cgatatttga agcgatagca aagattgaag gtttattata accatggtac
      901 gcgtgctaga ggcatcaaat aaaacgaaag gctcagtcga aagactgggc ctttcgtttt
      961 atctgttgtt tgtcggtgaa cgctctcctg agtaggacaa atccgccgcc ctagacctag
     1021 ggcgttcggc tgcggcgagc ggtatcagct cactcaaagg cggtaatacg gttatccaca
     1081 gaatcagggg ataacgcagg aaagaacatg tgagcaaaag gccagcaaaa ggccaggaac
     1141 cgtaaaaagg ccgcgttgct ggcgtttttc cataggctcc gcccccctga cgagcatcac
     1201 aaaaatcgac gctcaagtca gaggtggcga aacccgacag gactataaag ataccaggcg
     1261 tttccccctg gaagctccct cgtgcgctct cctgttccga ccctgccgct taccggatac
     1321 ctgtccgcct ttctcccttc gggaagcgtg gcgctttctc aatgctcacg ctgtaggtat
     1381 ctcagttcgg tgtaggtcgt tcgctccaag ctgggctgtg tgcacgaacc ccccgttcag
     1441 cccgaccgct gcgccttatc cggtaactat cgtcttgagt ccaacccggt aagacacgac
     1501 ttatcgccac tggcagcagc cactggtaac aggattagca gagcgaggta tgtaggcggt
     1561 gctacagagt tcttgaagtg gtggcctaac tacggctaca ctagaaggac agtatttggt
     1621 atctgcgctc tgctgaagcc agttaccttc ggaaaaagag ttggtagctc ttgatccggc
```

```
1681 aaacaaacca ccgctggtag cggtggtttt tttgtttgca agcagcagat tacgcgcaga
1741 aaaaaaggat ctcaagaaga tcctttgatc ttttctacgg ggtctgacgc tcagtggaac
1801 gaaaactcac gttaagggat tttggtcatg tctgtgcaag tactactgtt ctgcaggcgg
1861 atttgtccta ctcaggagag cgttcaccga caaacaacag ataaaacgaa aggcccagtc
1921 tttcgactga gcctttcgtt ttatttgatg cctctagcac gcgtaccatg ggatccccca
1981 tcaagcttta agacccactt tcacatttaa gttgtttttc taatccgcat atgatcaatt
2041 caaggccgaa taagaaggct ggctctgcac cttggtgatc aaataattcg atagcttgtc
2101 gtaataatgg cggcatacta tcagtagtag gtgtttccct ttcttcttta gcgacttgat
2161 gctcttgatc ttccaatacg caacctaaag taaaatgccc cacagcgctg agtgcatata
2221 atgcattctc tagtgaaaaa ccttgttggc ataaaaaggc taattgattt tcgagagttt
2281 catactgttt ttctgtaggc cgtgtaccta aatgtacttt tgctccatcg cgatgactta
2341 gtaaagcaca tctaaaactt ttagcgttat tacgtaaaaa atcttgccag ctttcccctt
2401 ctaaagggca aaagtgagta tggtgcctat ctaacatctc aatggctaag gcgtcgagca
2461 aagcccgctt atttttttaca tgccaataca atgtaggctg ctctacacct agcttctggg
2521 cgagtttacg ggttgttaaa ccttcgattc cgacctcatt aagcagctct aatgcgctgt
2581 taatcacttt acttttatct aatctagaca tggtaccttt ctcctcttta atgaattcgg
2641 tcagtgcgtc ctgctgatgt gctcagtatc tgctaaaccg gtttaataac tcgaggtgaa
2701 gacgaaaggg cctcgtgata cctagtgctt ggattctcac caataaaaaa cgcccggcgg
2761 caaccgagcg ttctgaacaa atccagatgg agttctgagg tcattactgg atctatcaac
2821 aggagtccaa gcgagctcga tatcaaatta cgccccgccc tgccactcat cgcagtactg
2881 ttgtaattca ttaagcattc tgccgacatg gaagccatca cagacggcat gatgaacctg
2941 aatcgccagc ggcatcagca ccttgtcgcc ttgcgtataa tatttgccca tggtgaaaac
3001 gggggcgaag aagttgtcca tattggccac gtttaaatca aaactggtga aactcaccca
3061 gggattggct gagacgaaaa acatattctc aataaaccct ttagggaaat aggccaggtt
3121 ttcaccgtaa cacgccacat cttgcgaata tatgtgtaga aactgccgga aatcgtcgtg
3181 gtattcactc cagagcgatg aaaacgtttc agtttgctca tggaaaacgg tgtaacaagg
3241 gtgaacacta tcccatatca ccagctcacc gtctttcatt gccatacgaa attccggatg
3301 agcattcatc aggcgggcaa gaatgtgaat aaaggccgga taaaacttgt gcttattttt
3361 ctttacggtc tttaaaaagg ccgtaatatc cagctgaacg gtctggttat aggtacattg
3421 agcaactgac tgaaatgcct caaaatgttc tttacgatgc cattgggata tatcaacggt
3481 ggtatatcca gtgatttttt tctccatttt agcttcctta gctcctgaaa atctcgataa
3541 ctcaaaaaat acgcccggta gtgatcttat ttcattatgg tgaaagttgg aacctcttac
3601 gtgccgatca acgtctcatt ttcgccagat atcgacgtct aagaaaccat tattatcatg
3661 acattaacct ataaaaatag gcgtatcacg aggccctttc gtcttcac
//
```
