# Supplementary material for: A GCDGC-specific DNA (cytosine-5) methyltransferase that methylates the GCWGC sequence on both strands and the GCSGC sequence on one strand
Source: PLoS One. 2022 Mar 21;17(3):e0265225. doi: 10.1371/journal.pone.0265225 (PMC8936443; doi:10.1371/journal.pone.0265225)
Supplement: S1 Raw images — (PDF) [file pone.0265225.s008.pdf]

A

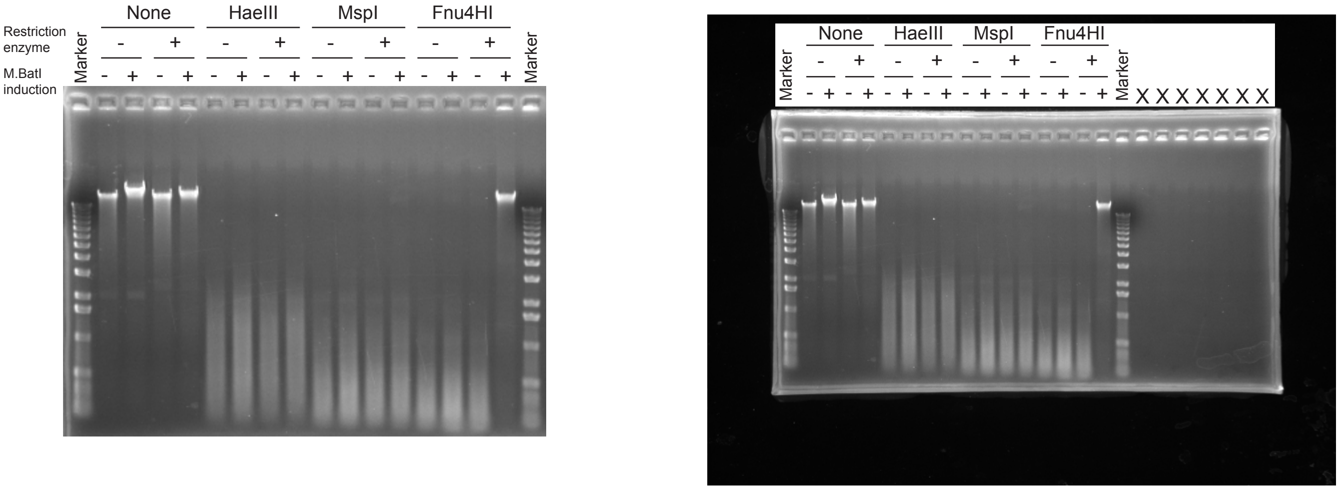

B

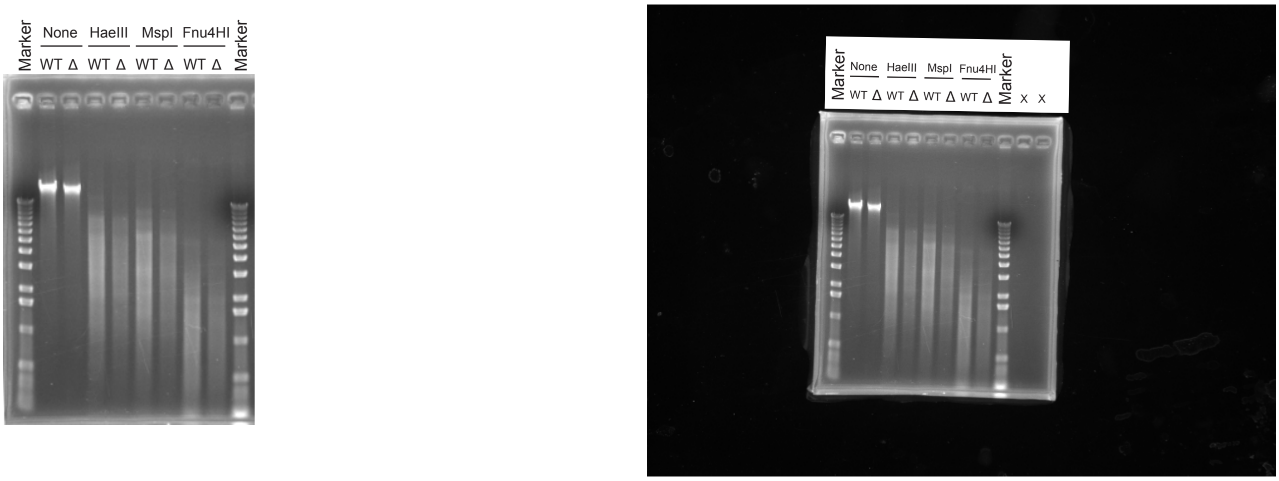

Fig 2. Adjusted figures are on the left and their raw images are on the right. Gel images were captured by AE-6933 Printgraph system (ATTO, Tokyo, Japan).

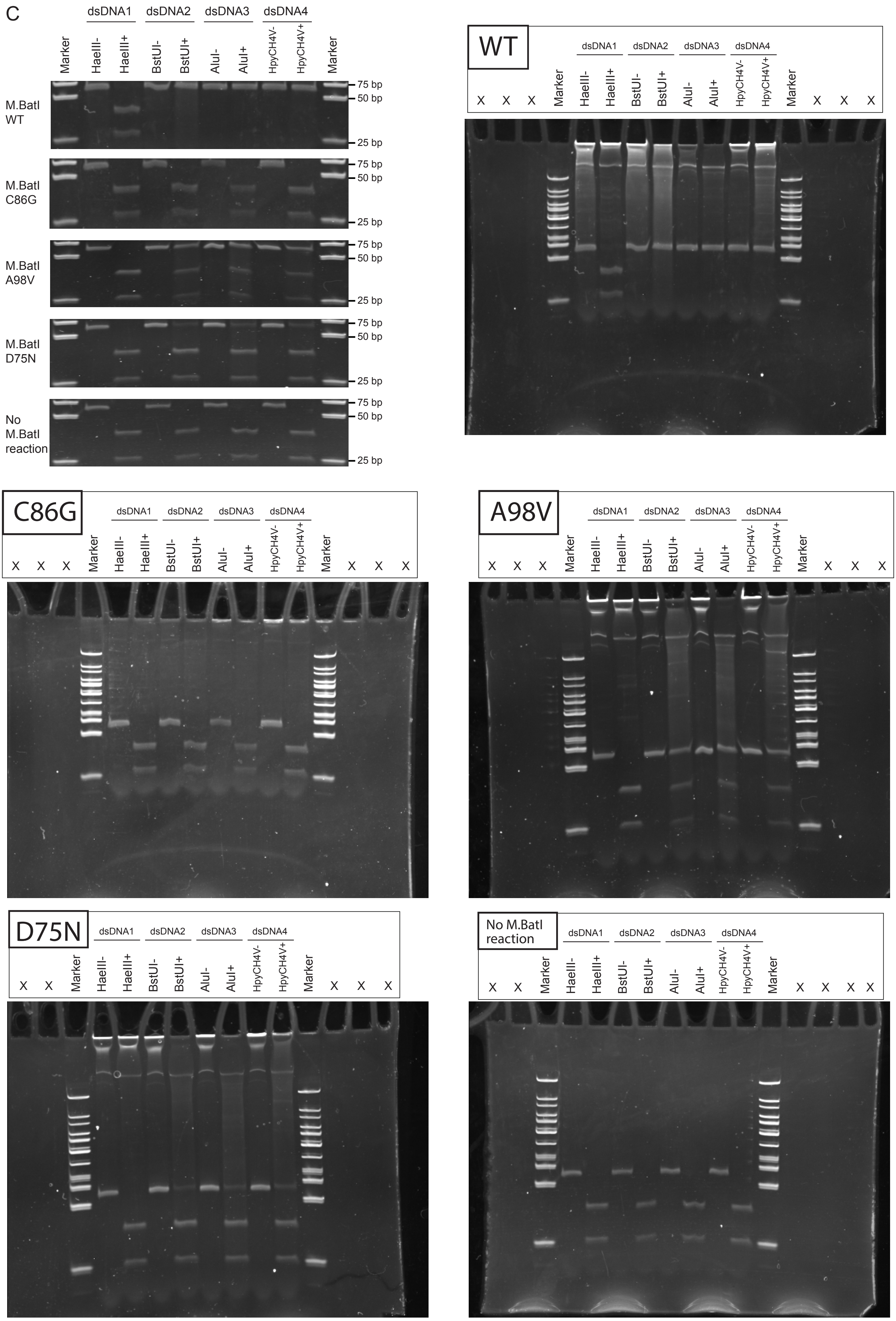

Fig 3. Adjusted figure is on the top left and its raw images are shown. Gel images were captured by AE-6933 Printgraph system (ATTO, Tokyo, Japan).

A

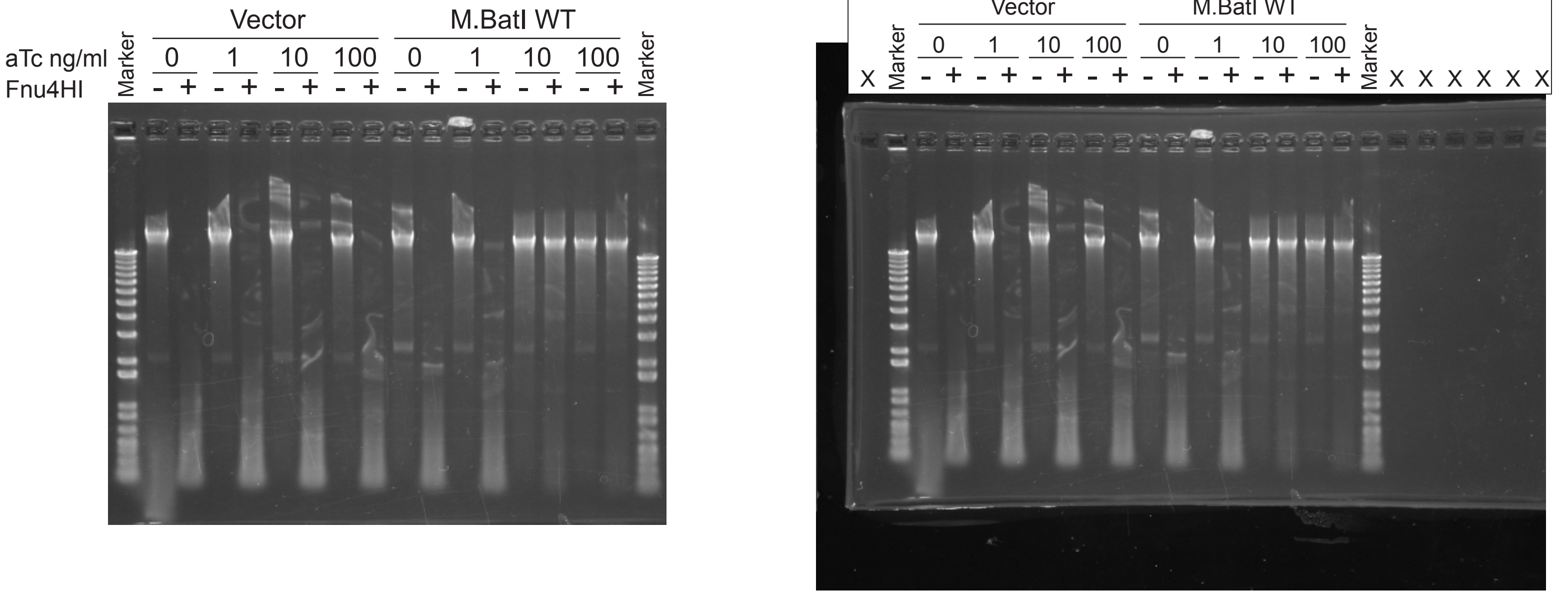

B

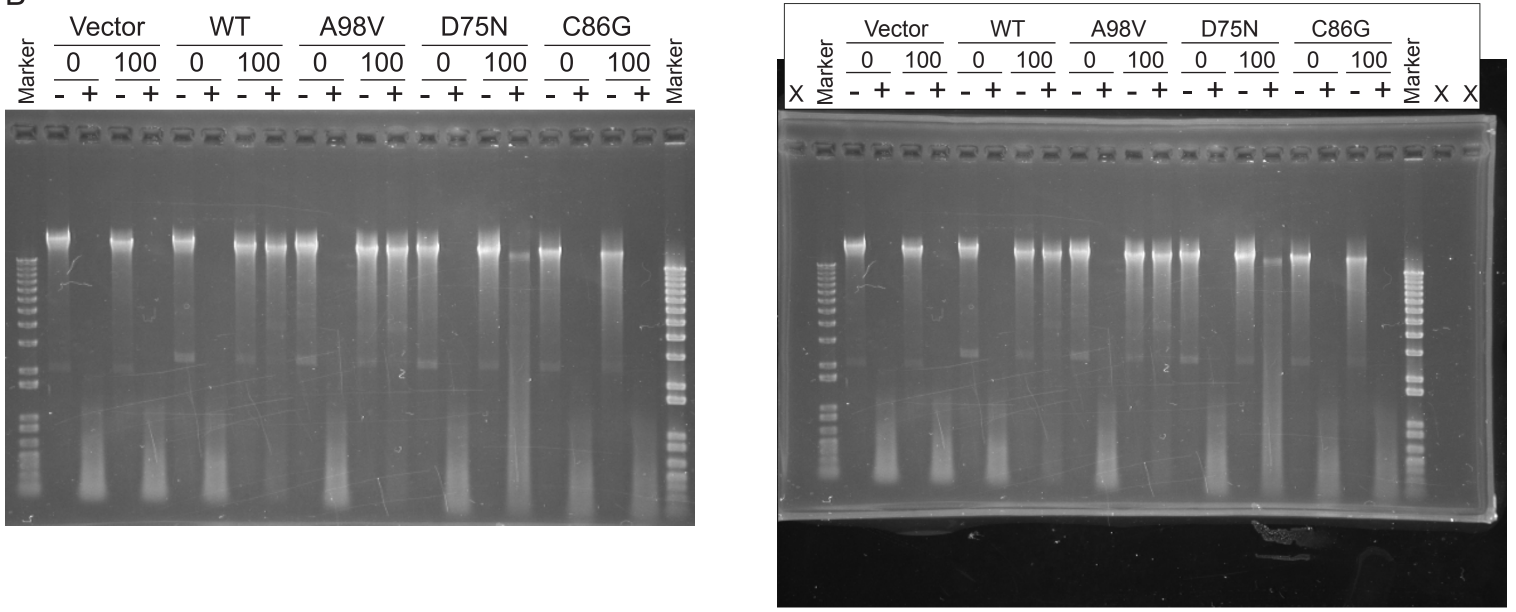

Fig 5. Adjusted figures are on the left and their raw images are on the right. Gel images were captured by AE-6933 Printgraph system (ATTO, Tokyo, Japan).

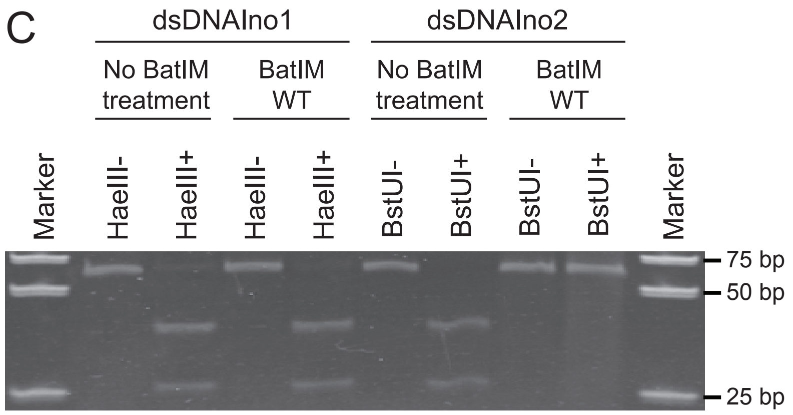

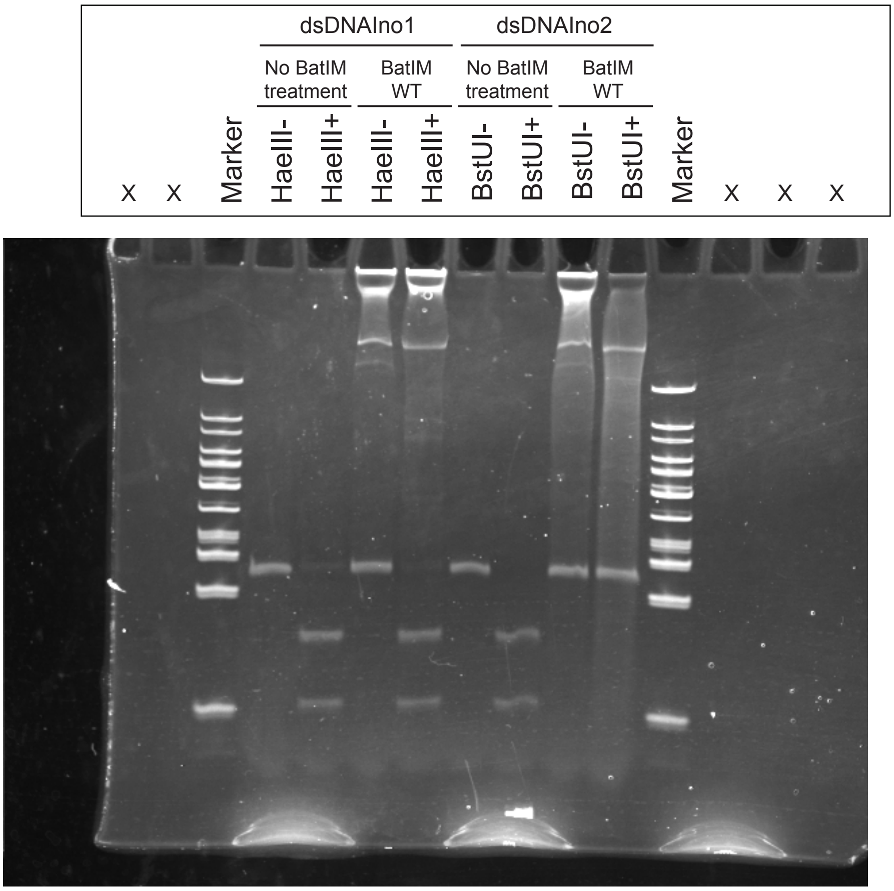

S3 Fig. Adjusted figures is on the top and its raw image is at the bottom. Gel images were captured by AE-6933 Printgraph system (ATTO, Tokyo, Japan).
